# Association of Apolipoprotein C3 Genetic Polymorphisms with the Risk of Ischemic Stroke in the Northern Chinese Han Population

**DOI:** 10.1371/journal.pone.0163910

**Published:** 2016-09-30

**Authors:** Yanzhe Wang, Xiaoyu Yin, Lei Li, Shumin Deng, Zhiyi He

**Affiliations:** Department of Neurology, The First Affiliated Hospital of China Medical University, Shenyang, 110001, China; National Cancer Center, JAPAN

## Abstract

The apolipoprotein C3 (APOC3) gene, which is a member of the APOA1/C3/A4/A5 gene cluster, plays a crucial role in lipid metabolism. Dyslipidemia is an important risk factor for ischemic stroke. In the present study, we performed a hospital-based case—control study of 895 ischemic stroke patients and 883 control subjects to examine the effects of four APOC3 single nucleotide polymorphisms (SNPs) (rs2854116, rs2854117, rs4520 and rs5128) on the risk of ischemic stroke in a northern Chinese Han population. The SNaPshot Multiplex sequencing assay was used for SNP genotyping, and the potential association of genotype distributions and allele frequencies with ischemic stroke was analyzed statistically. Compared with the GG genotype, the CC+GC genotype of rs5128 was significantly associated with an increased risk in females (adjusted OR = 3.38, 95% CI = 1.82–6.28, P <0.01) after all of the risk factors were adjusted for with logistic regression analyses. A similar relationship was found between the rs4520 polymorphism and ischemic stroke risk in Han Chinese women. Under a recessive genetic model, the TT+TC genotypes of this variant increased ischemic stroke risk (adjusted OR = 2.05; 95% CI = 1.28–3.29; P <0.01). Haplotype analysis revealed that in males, the T-C-T-C haplotype of rs2854116-rs2854117-rs4520-rs5128 was significantly more frequent in the ischemic stroke group than in the control group (OR = 1.49, 95% CI = 1.18–1.87, P<0.01). The results of our study indicate that the APOC3 polymorphisms contribute to ischemic stroke susceptibility in females in the northern Chinese Han population.

## Introduction

Stroke is a major cause of adult disability and death worldwide [1,2]. Ischemic stroke, which is the most common form, occurs as a result of local cerebral ischemia due to an obstruction within a brain-supplying blood vessel. Given that ischemic stroke-induced disability increases the social economic burden, outstanding strategies for prevention and for early detection in high-risk populations are key to handling this global issue; such strategies not only decrease morbidity by directing at-risk individuals into disease-monitoring networks but also provide the benefit of increasing the likelihood that reperfusion treatment can be administered within the therapeutic window when a stroke occurs [3–5].

Because ischemic stroke is a complex disease that involves many risk factors, including genetic variants and environmental factors, researchers have emphasized the search for genetic factors that increase ischemic stroke risk to develop novel and effective approaches to identify high-risk groups. To date, large-scale genome-wide association studies (GWAS) have identified several genetic polymorphisms that increase the risk for ischemic stroke [6]. However, these single nucleotide polymorphisms (SNPs) have a limited effect and explain only a small proportion of the heritability of ischemic stroke [7]. Therefore, additional SNPs for ischemic stroke need to be identified.

Dyslipidemia is an important risk factor for ischemic stroke, and genes involved in dyslipidemia might be candidate genes for ischemic stroke susceptibility. The apolipoprotein C3 (APOC3) gene, which is a member of the APOA1/C3/A4/A5 gene cluster and is located on chromosome 11q23, plays a crucial role in lipid metabolism [8,9]. APOC3 encodes a 79-amino-acid glycoprotein that is produced mainly in the liver, where it inhibits the action of lipoprotein lipase and interferes with receptor-mediated lipoprotein uptake. Four common SNPs in the APOC3 gene, rs2854116, rs2854117, rs4520 and rs5128, have been identified as putative functional SNPs that influence serum APOC3 concentrations and result in dyslipidemia by influencing triglyceride (TG) and very-low-density lipoprotein (VLDL) levels [10–13]. Therefore, SNPs in the APOC3 gene may affect ischemic stroke susceptibility. The association of APOC3 SNPs with coronary artery disease has been reported, but the findings have been inconclusive [14,15]. To the best of our knowledge, no study has investigated the association between APOC3 and ischemic cerebrovascular disease.

In the present study, we performed a hospital-based case—control study of 895 ischemic stroke patients and 833 control subjects to examine the effect of four SNPs (rs2854116, rs2854117, rs4520 and rs5128) of APOC3 on the risk of ischemic stroke in a northern Chinese Han population.

## Materials and Methods

### Study Population

The case—control study included 895 patients and 883 healthy, age- and sex-matched controls. The patients were collected from the Department of Neurology at the First Affiliated Hospital of China Medical University between December 2013 and December2015. All of the subjects were 40 to 80 years old. Patients were considered eligible if they were receiving their first diagnosis of acute ischemic stroke based on neurological examination and radiological imaging; such diagnoses included the sudden onset of focal neurological deficit that persisted for more than 24 h with corresponding infarction on brain imaging (computed tomography and magnetic resonance imaging). Patients with transient ischemic attack, cadioembolism, cerebral trauma, cerebrovascular malformations, coagulation disorders, autoimmune diseases, tumors, or chronic infectious diseases were excluded. Cases with renal or liver diseases, hematopathy, occlusive arterial disease or phlebothrombosis of the limbs were also excluded. The controls were recruited from the physical examination department of the First Affiliated Hospital of China Medical University and lacked evidence of stroke or other neurological diseases. In the control group, individuals with tumors, autoimmune diseases, liver ailments, nephrosis or hematological diseases were excluded. All subjects were members of the Han population living in the Liaoning province of northern China. Clinical records, including the general condition (gender, age, body mass index (BMI), ethnicity, history of smoking and alcohol use), past history (history of diabetes and hypertension), family history of stroke, serum TG, total cholesterol (TC), low-density lipoprotein (LDL), and high-density lipoprotein (HDL) were collected for the patients and controls. Brain CT or MRI, echocardiography, carotid ultrasound, transcranial Doppler, and electrocardiograms were also required for all patients. BMI was categorized based on the Asian classification of obesity, BMI >22.9 considered overweight. Hyperlipidemia was defined as a total plasma cholesterol level of 5.72 mmol/L and/or plasma TG level of 1.7 mmol/L or current use of lipid-lowering drugs. This study was approved by the Institutional Ethical Committee of the First Affiliated Hospital of China Medical University, and was conducted according to The Code of Ethics of the World Medical Association (Declaration of Helsinki). Written informed consent was obtained from all of the participants.

### DNA Extraction and Genotyping

Genomic DNA was extracted from 200 μL of EDTA-anticoagulated peripheral blood using a DNA Purification Kit (Promega, Madison, USA). The DNA purity was measured with spectrophotometry, and the DNA samples were stored at −80°C. The genotypes were analyzed using the SNaPshot Multiplex Kit (Applied Biosystems Co., Ltd., Foster City, CA, USA). The SNaPshot reactions were executed in a final volume of 10 μL, including 5 μL of the SNaPshot Multiplex Kit (ABI), 1 μL primer mix, 2 μL water, and 2 μL templates, which consisted of the multiplex PCR products for the different genes. The SNaPshot reaction procedures were as follows: (1) initial denaturation at 96°C for 1 min; (2) denaturation at 96°C for 10 s; (3) annealing at 55°C for 5 s; and (4) extension at 60°C for 30 s, for a total of 28 cycles. The amplified samples were stored at 4°C. The extension products were purified through a 1-h incubation with 1 U of shrimp alkaline phosphatase (Takara: Otsu, Shiga, Japan) at 37°C, followed by incubation at 75°C for 15 min to inactivate the enzyme. The purified products (0.5 μL) were mixed with 9 μL of Hi—Di and 0.5 μL of the Liz120 size standard (Applied Biosystems Co., Ltd.). The samples were incubated at 95°C for 5 min and then loaded onto an ABI 3130XL DNA sequence detector for capillary electrophoresis. The experimental results were analyzed with GeneMapper 4.0 (Applied Biosystems Co., Ltd.).

### Statistical Analysis

All statistical analyses were performed with Statistical Product and Service Solutions (SPSS) v20.0 if not otherwise specified. All of the tests were two-sided, and statistical significance was defined as P<0.05. Pearson’s chi-square test was used to compare the distribution of the demographic variables and to examine differences in risk factors and genotypes and in alleles and haplotypes between the cases and controls. For each genotype, Hardy-Weinberg equilibrium (HWE) was tested with a goodness-of-fit χ2 test. Unconditional logistic regression was performed to calculate the odds ratios (ORs) and 95% confidence intervals (CIs) to estimate the association between certain genotypes and ischemic stroke. The Quanto power calculator was used to calculate power in our cohort. With a sample size of 895 cases and 833 controls, and assuming a genotypic relative risk for the recessive model of 2, an MAF of 0.25, a population prevalence of ischemic stroke of 1.88%, and a Type I error probability of 0.05, we will be able to reject the null hypothesis of an odds ratio equals to 1 with a probability (power) of 98.24%. Based on the observed frequencies of the four SNPs, we used the SHEsis analysis platform to calculate a linkage disequilibrium index (D’ and r^2^) and infer haplotype frequencies. [16,17]

## Results

The general characteristics of the 895 ischemic stroke patients and 883 controls are summarized in Table 1. There was no significant difference in age or gender between the normal controls and the patients. However, conventional risk factors for ischemic stroke, such as BMI, diabetes mellitus, hypertension, history of smoking, history of alcohol use and hyperlipidemia were significantly more common in the ischemic stroke patient group than in the control group.

**Table 1:** Characteristics and risk factors for stroke.

Variable	Cases n (%)	Controls n (%)	P value
Age (years)	64.48±8.51	63.70±6.68	0.33
Age (≤60/>60)	319(35.6)/576(64.4)	288(32.6)/595(67.4)	0.18
Gender (male/female)	499(55.8)/396(44.2)	457(51.8)/426(48.2)	0.09
BMI (≤22.9/>22.9)	454(50.7)/441(49.3)	274(65.0)/148(35.0)	<0.01
Diabetes mellitus	234(26.1)	64(7.2)	<0.01
Hypertension	552(61.7)	178(20.2)	<0.01
History of smoking	310(34.6)	137(15.5)	<0.01
History of alcohol use	154(17.2)	98(11.1)	<0.01
family history of stroke	63(7.0)	46(5.2)	0.11
Hyperlipidemia	324(36.2)	177(20.0)	<0.01

All of the allele distributions were consistent with HWE (rs2854116: P = 0.10; rs2854117: P = 0.14; rs4520: P = 0.06; rs5128: P = 0.28). Because the association between ischemic stroke and APOC3 alleles may be affected by endogenous sex hormones in a sexually dimorphic manner, a subtype analysis was performed on the genotype and allele frequencies of the four SNPs among the 895 ischemic stroke patients and the 883 control subjects, as shown in Tables 2 and 3.

**Table 2:** Allele and genotype frequencies of genetic polymorphisms among cases and controls as well as their main effects on stroke risk in the male population.

SNP	Cases	Percent	Controls	Percent	OR(95% CI) * ^a^	P value ^b^
rs2854116						
TT (ref)	175	35.10%	155	33.90%	1.00 (ref)	-
TC	219	43.90%	219	47.90%	1.00(0.61–1.65)	0.99
CC	105	21.00%	83	18.20%	1.45(0.67–3.14)	0.35
Dominant model TC+CC vs TT					1.08(0.66–1.76)	0.76
Recessive model TT+TC vs CC					0.69(0.36–1.33)	0.27
rs2854117						
CC (ref)	172	34.50%	174	38.10%	1.00 (ref)	-
CT	220	44.10%	207	45.30%	1.33(0.81–2.20)	0.26
TT	107	21.40%	76	16.60%	1.37(0.64–2.90)	0.42
Dominant model CT+TT vs CC					1.50(0.94–2.40)	0.09
Recessive model CC+CT vs TT					0.98(0.50–1.90)	0.95
rs4520						
TT (ref)	182	36.50%	147	30.40%	1.00 (ref)	-
TC	242	48.50%	258	58.20%	0.74(0.53–1.04)	0.82
CC	75	15.00%	52	11.40%	1.10(0.64–1.90)	0.73
Dominant model TC+CC vs TT					0.80(0.58–1.10)	0.17
Recessive model TT+TC vs CC					0.67(0.41–1.12)	0.13
rs5128						
CC (ref)	207	41.50%	170	37.20%	1.00 (ref)	-
CG	228	45.70%	228	49.20%	0.76(0.48–1.20)	0.24
GG	64	12.80%	62	13.60%	0.64(0.35–1.16)	0.14
Dominant model CG+GG vs CC					0.66(0.44–1.00)	0.05
Recessive model CC+CG vs GG					1.19 (0.71–2.01)	0.52

Abbreviations: SNP, single nucleotide polymorphism; OR, odds ratio; CI, confidence interval.

*ORs and 95% CIs were calculated by logistic regression.

^a,b^ Adjusted OR(95%CI) and P value, adjusted for age, gender, BMI, diabetes mellitus, hypertension, history of smoking, history of alcohol use, family history of stroke and hyperlipidemia.

**Table 3:** Allele and genotype frequencies of genetic polymorphisms among cases and controls as well as their main effects on stroke risk in the female population.

SNP	Cases	Percent	Controls	Percent	OR(95% CI) * ^a^	P value ^b^
rs2854116						
TT (ref)	131	33.1%	109	25.6%	1.00 (ref)	-
TC	186	47.0%	219	51.4%	1.05(0.53–2.11)	0.88
CC	79	19.9%	98	23.0%	1.59(0.50–5.00)	0.43
Dominant model TC+CC vs TT					1.11(0.56–2.19)	0.76
Recessive model TT+TC vs CC					0.66(0.24–1.81)	0.42
rs2854117						
CC (ref)	136	34.50%	111	26.10%	1.00 (ref)	-
CT	188	47.50%	226	53.10%	0.86(0.44–1.69)	0.66
TT	72	18.20%	89	20.90%	1.10(0.34–3.53)	0.88
Dominant model CT+TT vs CC					0.89(0.46–1.73)	0.73
Recessive model CC+CT vs TT					0.78(0.28–2.17)	0.64
rs4520						
TT (ref)	140	35.40%	196	46.00%	1.00 (ref)	-
TC	182	46.00%	132	31.00%	1.24(0.79–1.94)	0.34
CC	74	18.70%	98	23.00%	0.61(0.36–1.03)	0.07
Dominant model TC+CC vs TT					1.32(0.90–1.94)	0.16
Recessive model TT+TC vs CC					**2.05(1.28–3.29)**	**<0.01**
rs5128						
CC (ref)	177	44.70%	149	35.970%	1.00 (ref)	-
CG	178	44.90%	182	42.70%	0.67(0.44–1.05)	0.08
GG	41	10.40%	95	22.30%	**0.20(0.09–0.43)**	**<0.01**
Dominant model CG+GG vs CC					0.71(0.47–1.08)	0.11
Recessive model CC+CG vs GG					**3.38 (1.82–6.28)**	**<0.01**

Abbreviations: SNP, single nucleotide polymorphism; OR, odds ratio; CI, confidence interval.

*ORs and 95% CIs were calculated by logistic regression.

^a,b^ Adjusted OR(95%CI) and P value, adjusted for age, gender, BMI, diabetes mellitus, hypertension, history of smoking, history of alcohol use, family history of stroke and hyperlipidemia.

As shown in Table 2, none of the genotype and allele frequencies of the rs2854116, rs2854117, rs4520 and rs5128 APOC3 variants significantly differed between the male ischemic stroke patients and healthy controls. Moreover, no significant differences were observed between the ischemic stroke patients and the controls in Table 2. Moreover, no significant differences were observed between the male ischemic stroke male patients and controls in either the dominant or recessive model (Table 2).

In the female population, individuals with the homozygous GG genotype experienced a decreased risks of ischemic stroke compared to those with the homozygous wild-type CC genotype (adjusted OR = 0.20, 95% CI = 0.09–0.43, P <0.01) for rs5128. In the recessive model, we found that the CC+CG genotype of rs5128 was associated with a significantly increased risk of ischemic stroke compared with the homozygous wild-type CC genotype (adjusted OR = 3.38, 95% CI = 1.82–6.28, P <0.01). While the genotype and allele frequencies of rs4520 were similar between the ischemic stroke patients and controls, significant associations were observed for rs4520 in the recessive model. A higher frequency of the rs4520 TT+TC genotype was observed only among females with ischemic stroke; the TT+TC genotype was therefore positively correlated with the risk of ischemic stroke in the female subgroup (adjusted OR = 2.05; 95% CI = 1.28–3.29; P <0.01) (Table 3).

The plasma lipid levels of individuals with the CC, CG, GG genotypes of rs5128 and the TT, TC, CC genotypes of rs4520 are listed in Table 4, statistical analysis of these results did not reveal any significant difference (P>0.05).

**Table 4:** Plasma lipid levels among different individuals with various rs5128 genotypes and rs4520 genotypes.

	rs5128				rs4520			
	CC	CG	GG	P value	TT	TC	CC	P value
TG (mmol/L)	1.63±0.79	1.62±0.85	1.60±0.72	0.18	1.60±0.78	1.66±0.87	1.57±0.68	0.87
TC (mmol/L)	4.89±1.08	4.87±1.03	4.80±1.02	0.97	4.86±1.01	4.84±1.06	4.87±1.12	0.49
HDL (mmol/L)	1.38±0.36	1.36±0.36	1.36±0.30	0.81	1.37±0.33	1.36±0.36	1.38±0.35	0.36
LDL (mmol/L)	2.80±0.70	2.76±0.89	2.90±0.94	0.15	2.82±0.90	2.81±0.89	2.70±0.82	0.07

The four SNPs were in linkage disequilibrium in this study population (D'>0.8 or r^2^>0.4). Of all the possible haplotypes, only 5 had a frequency of >0.03 among both cases and controls and were included in the haplotype analysis. These five haplotypes represented 91.3% of the chromosomes of the cases and 84.6% of the controls among males, and 91.7% of the chromosomes of the cases and 87.1% of the controls among females. There was no difference in the overall haplotype distribution between the cases and controls. The subtype analysis revealed that the frequency of the T-C-T-C haplotype in the patients was significantly higher than that in the controls, but just among females (OR = 1.489, 95% CI = 1.18–1.87, P<0.01) (Table 5).

**Table 5:** Haplotype results for males and females.

Haplotype	Males				Females			
	Cases (%)	Controls (%)	OR (95% CI)	P value	Cases (%)	Controls (%)	OR (95% CI)	P value
T-C-T-C	264(26.4)	212(23.1)	1.18(0.96–1.46)	0.12	221(27.9)	177(20.8)	1.49(1.18–1.87)	<0.01
T-C-T-G	35(3.5)	60(6.5)	0.51(0.33–0.78)	<0.01	29(3.6)	35(4.1)	0.89(0.54–1.47)	0.64
T-C-C-C	230(23.1)	204(22.3)	1.03(0.83–1.28)	0.76	172(21.7)	163(19.2)	1.18(0.92–1.50)	0.19
C-T-T-G	278(27.8)	224(24.5)	1.18(0.96–1.45)	0.12	192(24.3)	273(32.0)	0.68(0.54–0.84)	<0.01
C-T-C-C	104(10.5)	73(8)	1.33(0.97–1.82)	0.08	112(14.1)	94(11.1)	1.33(0.99–1.78)	0.06

Abbreviations: OR, odds ratio; CI, confidence interval.

## Discussion

In the present study, we examined the effects of four APOC3 polymorphisms (rs2854116, rs2854117, rs4520 and rs5128) on the risk of ischemic stroke in a northern Chinese Han population. Surprisingly, after multivariate adjustment for conventional risk factors of stroke, the CC+GC genotype were significantly associated with an increased risk of ischemic stroke in the northern Chinese Han population, but only in females. Similar results were found regarding the relationship between the rs4520 polymorphism and the ischemic stroke risk of Chinese Han women. Under a recessive genetic model, the genotypes for this variant (TT+TC) were associated with a higher ischemic stroke risk. We also predicted a higher risk of ischemic stroke for the T-C-T-C haplotype in northern Chinese Han females. To our knowledge, this is the first study to identify the association of APOC3 SNP with ischemic stroke risk in the northern Chinese Han population.

GWAS have demonstrated that common mutations within the APOC3 gene are associated with higher TG and many other diseases related to hyperlipidemia [18]. The functional promoter SNPs rs2854116 and rs2854117 were reported to be closely associated with acute coronary syndrome, and the minor allele of these two SNPs increased the risk of ischemic heart disease [11,19,20]. A previous case—control study suggested that rs4520 was associated with an increased risk of diabetes, probably through mechanisms other than direct effects on TG [21]. Studies have also suggested that rs5128 might contribute to an increased risk of coronary artery disease due to its effect on TG and VLDL metabolism [12]. Another study showed that the G allele of rs5128 might contribute to an increased risk of intracerebral hemorrhage due to its effect on serum lipid levels [10]. In line with the observations of the relationships between APOC3 SNPs and the disease described above, our study revealed the direct genetic effect of APOC3 on the risk of ischemic stroke. Importantly, the CC+CG genotype of rs5128 and the TT+TC genotype of rs4520 remained independent risk factors for ischemic stroke in northern Chinese Han females after adjusting for hyperlipidemia and other confounding factors. However, the rs5128 and rs4520 polymorphisms were clear independent risk factors for ischemic stroke in a manner that did not result from their effect on serum lipid levels.

In contrast, neither the genotype nor the allele frequencies of the APOC3 SNPs significantly differed between the male ischemic stroke patients and controls. Our findings indicate some degree of gender specificity in the effect of APOC3 genetic variation on ischemic stroke. This phenomenon might be explained, at least in part, by the presence of endogenous sex hormones such as estrogen, which mediate cholesterol metabolism and differ between men and women [22]. The majority of females are pre-menopausal and thus may benefit from the protective effects of estrogen on cerebrovascular risk [23]. Moreover, by regulating expression of target gene, hormone-induced DNA methylation may increase or reduce the risk of complex diseases such as ischemic stroke [24]. Recently, researchers have determined the methylation patterns of the APOC3 gene that may be directly involved in transcriptional regulation of this gene [25]. Therefore, the gender difference in the effect of APOC3 genetic variation on ischemic stroke is likely attributable to epigenetic modifications such as DNA methylation. To the best of our knowledge, this study is the first attempt to report a gender-specific association of APOC3 polymorphisms with ischemic stroke. Nevertheless, our results for ischemic stroke differ from those of previous studies of ischemic vascular disease [11,19]. The disagreement between the results of our study and those of previous studies could be due to differences in study design, sample size, and sample selection as well as in the genetic background and geographical and ethnic origins of the populations studied.

The exact mechanism through which the CC+CG genotype of rs5128 and the TT+TC genotype of rs4520 might contribute to the risk of ischemic stroke is unclear. Rs5128, which is also known as the Sst I polymorphism, is located in the 3’-untranslated region of the APOC3 gene. Rs5128 might facilitate microRNA binding and change the transcriptional activity of APOC3, leading to higher plasma APOC3 levels and dyslipidemia [26,27]. Furthermore, we demonstrated through haplotype analysis that rs5128 and rs4520 are in linkage disequilibrium with rs2854116 and rs2854117, which are located in an insulin response element (IRE) within the APOC3 promoter. Polymorphisms in the IRE have recently been shown to render the gene unresponsive to normal transcriptional repression through peroxisome proliferator-activated receptors (PPARs) [28], which lower serum TGs and influence lipid metabolism. It is rational to hypothesize that the mechanism through which the rs5128 and rs4520 polymorphisms increase ischemic stroke risk may involve an inhibitory effect on PPAR-mediated transcriptional repression. Additionally, in both transfected McA-RH7777 cells under lipid-rich conditions and in high-fat-diet-fed APOC3 knockout mice in which hepatic APOC3 expression was restored, the impaired lipid binding of mutant APOC3 was disabled, promoting VLDL assembly and secretion [29]. Therefore, APOC3 plays an essential role in lipid metabolism, and this role could explain the potential association of rs4520 and rs5128 with ischemic stroke.

However, our study has some shortcomings that should be recognized. The most limitation is the sample size, which was not particularly large. In addition, we did not evaluate the reproducibility of the results because collecting thousands of eligible northern Chinese Han individuals is very time-consuming. We are preparing a replication study of a larger sample size (the second stage, another case-control population) to confirm the results. Second, our analysis only investigated the northern Chinese Han population, and whether these results can be applied to other ethnic populations remains unclear. Nevertheless, potential confounding factors were minimized by matching age, sex, and ethnicity. Third, there are limitations related to the study design in that a retrospective case—control study cannot confirm a cause—effect relationship. Our study should also be considered preliminary due to the lack of measurements of APOC3 mRNA and plasma APOC3 concentrations. The results need to be confirmed with more detailed expression and translational studies. Finally, ischemic stroke is a complex disease, the occurrence of which is related to multiple genes and environmental factors that were not evaluated in the present study.

In conclusion, the results of our study indicate that the APOC3 polymorphisms were significantly associated with the risk of ischemic stroke in the northern Chinese Han population. We provided the novel insight that the CC+GC genotype of rs5128 and the TT+TC genotype of rs4520 contributed to female-specific ischemic stroke susceptibility in the northern Chinese Han population. Detecting APOC3 polymorphisms may increase awareness of the risk of ischemic stroke, and individuals in high-risk groups might be advised to receive regular checkups to reduce the adverse effects of ischemic stroke.

## Supporting Information

S1 Dataset Full dataset. All data in this article is available in S1 Dataset.
(SAV)
